# GDF15 associates with, but is not responsible for, exercise-induced increases in corticosterone and indices of lipid utilization in mice

**DOI:** 10.1152/japplphysiol.00519.2024

**Published:** 2024-10-31

**Authors:** Meagan Arbeau, Bradley J. Baranowski, Stewart Jeromson, Annalaura Bellucci, Michael Akcan, Serena Trang, Katelyn Eisner, Kyle D. Medak, David C. Wright

**Affiliations:** ^1^School of Kinesiology, University of British Columbia, Vancouver, British Columbia, Canada; ^2^British Columbia Children’s Hospital Research Institute, Vancouver, British Columbia, Canada; ^3^Deparment of Human Health and Nutritional Sciences, University of Guelph, Guelph, Ontario, Canada; ^4^Faculty of Land and Food Systems, University of British Columbia, Vancouver, British Columbia, Canada

**Keywords:** corticosterone, exercise, growth differentiation factor 15, mice, nutrition

## Abstract

Growth differentiation factor 15 (GDF15) is a stress-induced cytokine that increases with exercise and is thought to increase corticosterone and lipid utilization. How postexercise nutrient availability impacts GDF15 and the physiological role that GDF15 plays during and/or in the recovery from exercise has not been elucidated. The purpose of this investigation was to examine how postexercise nutrient availability impacts GDF15 and to use this as a model to explore associations between GDF15, corticosterone, and indices of lipid and carbohydrate metabolism. In addition, we explored the causality of these relationships using GDF15-deficient mice. Male and female C57BL/6J mice ran for 2 hours on a treadmill and were euthanized immediately or 3 hours after exercise with or without access to a chow diet. In both sexes, circulating concentrations of GDF15, corticosterone, nonesterified fatty acids (NEFA), and beta-hydroxybutyrate (BHB) were higher immediately postexercise and remained elevated when food was withheld during the recovery period. While serum GDF15 was positively associated with corticosterone, BHB, and NEFA, increases in these factors were similar in wild-type and GDF15^−/−^ mice following exercise. The lack of a genotype effect was not explained by differences in insulin, glucagon, or epinephrine after exercise. Our findings provide evidence that while GDF15 is associated with increases in corticosterone and indices of lipid utilization this is not a causal relationship.

**NEW & NOTEWORTHY** Circulating growth differentiation factor 15 (GDF15) increases during exercise, but the physiological role that it plays has not been elucidated. Recent data suggest that GDF15 regulates corticosterone and lipid utilization. Here we demonstrate that postexercise nutrient availability influences GDF15 in the recovery from exercise and GDF15 is associated with corticosterone and indices of lipid utilization. However, the associations were not causal as exercise-induced increases in fatty acids, beta-hydroxybutyrate, and corticosterone were intact in GDF15^−/−^ mice.

## Graphical abstract

**Figure:**
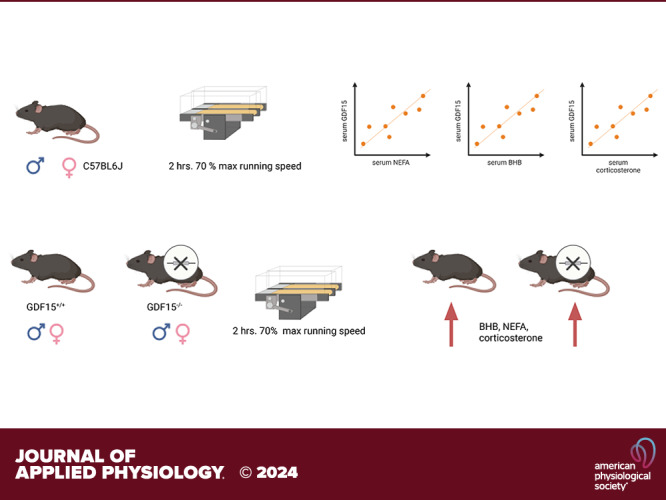

## INTRODUCTION

Growth differentiation factor 15 (GDF15) is a stress-induced cytokine and a member of the transforming growth factor-beta superfamily (1). GDF15 signals through a receptor that is exclusively expressed in the hindbrain called glial cell-derived neurotrophic factor family receptor-alpha-like (GFRAL) (2–5) and has well-characterized effects on energy balance. In this regard, GDF15 treatment in rodents reduces food intake (2–5) and the preference for foods higher in fat and sugar (6, 7), while more recent work has highlighted a role for GDF15 in the maintenance of energy expenditure during periods of caloric restriction (8).

In addition to being elevated in a number of pathological conditions such as mitochondrial disease (9), chemotherapy-induced muscle wasting (10), cardiovascular disease (11), heart failure (12), and obesity (1), circulating levels of GDF15 are acutely increased following exercise in rodents (13, 14) and humans (14–16), lasting for several hours into recovery (16). While the specific tissues, and underlying mechanisms, contributing to exercise-induced increases in GDF15 have not been fully elucidated, recent work has demonstrated that GDF15 is liver-derived and can be suppressed by insulin (16). Given these findings and the well-documented effects of nutrient ingestion on insulin, it seems reasonable to speculate that manipulating nutrition in the immediate postexercise period could impact GDF15.

The physiological role of acute increases in GDF15 with exercise (2–5) has not been delineated. Although pharmacological dosing of GDF15 reduces food intake, it does not appear to play a role in the control of energy intake following exercise. In support of this supposition, postexercise energy intake is not different between wild-type and GFRAL^−/−^ mice (14). Cimino et al. (17) reported that GDF15 activates the hypothalamic-pituitary-adrenal axis leading to increases in circulating corticosterone concentrations in mice. During exercise, increases in glucocorticoids (18–21) are thought to be an important signal (22) in the mobilization of fuel stores through stimulatory effects on liver glucose production (23) and adipose tissue lipolysis (24). Interestingly, the knockdown of GDF15 attenuates fasting-induced increases in circulating nonesterified fatty acids (NEFA) and beta-hydroxybutyrate (BHB), a marker of liver fatty acid oxidation (25). Taken together, these findings suggest that GDF15 could be playing a role in lipid mobilization during the recovery from exercise, potentially through a mechanism involving corticosterone.

In this investigation, we aimed to determine the impact of postexercise nutrient availability on GDF15 and to use this as a model to explore associations between GDF15 and indices of whole body fuel metabolism. Understanding the impact of postexercise nutrition on GDF15 could also provide insight into approaches that could be utilized to extend the potential beneficial metabolic effects of this hormone. We further wanted to examine if GDF15 was required for exercise-induced increases in corticosterone and markers of lipid utilization. We hypothesized that *1*) withholding food following exercise would prolong increases in GDF15; *2*) there would be positive associations between GDF15, corticosterone, and markers of lipid utilization; and *3*) GDF15 would be required for exercise-induced increases in corticosterone and indices of fatty acid metabolism.

## METHODS

### Animals and Ethics

All protocols were approved by the University of British Columbia Animal Care Committee (protocol no. A22-0021) and followed the Canadian Council on Animal Care Guidelines. Male and female C57BL/6J mice (∼16 wk of age; cat no. 000664; Jackson Laboratories) were group housed (∼4/cage) at room temperature (20–22°C) and given ad libitum access to standard chow (cat. no. 2918; Teklad) and water while acclimating to the animal facility at British Columbia Children’s Hospital Research Institute for ∼1 wk. As described previously (26), heterogenous breeding pairs of whole body GDF15^−/−^ mice were purchased from Taconic and backcrossed with C57BL/6J mice. GDF15^+/−^ mice were backcrossed to C57BL/6J mice for three generations and GDF15^−/−^ and wild-type (WT) littermates were used for the experiments. Mice were genotyped using primer sets described previously (26).

### Experimental Design

In the first set of experiments, we examined the impact of postexercise nutrition on GDF15. All treadmill exercise sessions occurred at the beginning of the murine dark cycle (∼6:00 PM), and mice in the sedentary group were moved into the same procedure room. Mice in the exercise groups were familiarized with a motorized rodent treadmill (Exer 3/6; Columbus Instruments) for 2 consecutive days for 15 min/day by running at 15 m/min on a 5% incline (27) and then given a rest period of ∼48 h before maximum running speed was assessed. Maximum speed was assessed by running mice at 10 m/min for 3 min at a 5% incline, treadmill speed was subsequently increased by 3 m/min every 3 min. Maximum speed was defined as the fastest speed mice could maintain for 3 continuous minutes.

Approximately 48 h following the maximum speed test, mice in the exercise groups ran for 2 h, or until exhaustion, at 70% of the predetermined maximum speed with an incline of 5% (16). Following exercise, mice were either euthanized immediately (exercise-0h), were single housed with ad libitum access to chow (exercise-chow; cat. no. 2918, Teklad; 58% carbohydrate, 18% fat, 24% protein by kcal, and 3.1 kcal/g), or had food withheld for 3 h (exercise-FW).

### Tissue Collection

Immediately following the sedentary, exercise or the respective feeding period, mice were anesthetized with pentobarbital (Euthansol; 120 mg/kg body wt, IP) and euthanized through exsanguination. Blood was collected via cardiac puncture, immediately placed on ice for ∼30 min before centrifugation (1,500 RCF for 10 min) and serum was collected. The heart, liver, inguinal white adipose (iWAT), and triceps muscles were harvested, snap frozen in liquid nitrogen, and stored at −80°C until further analysis. In the GDF15^−/−^ experiments, the treadmill exercise procedures and tissue collections were repeated; however, the terminal experiment included only three groups per genotype; sedentary, exercise-0h, and exercise-FW.

### Serum Analysis

All metabolite and hormone assays were analyzed using a Versamax Tunable Microplate Reader and SoftMax Pro Software (Molecular Devices). Serum GDF15 (cat. no. DY6385; R&D Systems), insulin (cat. no. 10-1247-01; Mercodia Inc.), corticosterone (cat. no. 55-CORMS-E01; Alpco), glucagon (cat. no. 10-1281-01; Mercodia), and epinephrine (cat. no. KA3768; Abnova) were measured with commercially available enzyme-linked immunosorbent assay (ELISA) kits. Serum glucose (cat. no. 10009582; Cayman Chemicals), NEFA (cat. no. CA97000-012; Wako Chemicals), and BHB (cat. no. 700190; Cayman Chemicals) were measured using commercially available colorimetric assay kits.

### Real-Time PCR

As described previously (13, 16, 28), *GDF15* mRNA expression was measured using real-time PCR. RNA was extracted from the liver, adipose tissue, and skeletal muscle using Trizol and Qiagen RNeasy Mini Kits (cat. no. 74106) followed by DNase-free treatment (Thermo Fisher Scientific; cat. no. AM1906) for the removal of genomic DNA. cDNA was synthesized using Superscript II (cat. no. 4368814; Thermo Fisher Scientific), and real-time PCR was run using SYBR Green Supermix (cat. no. 1725271; Bio-Rad) on a Bio-Rad CFX connect system. Expression of *Gdf15* was measured relative to the housekeeping gene, *Ppib* (29), using the 2^−ΔΔCT^ method (30). *Ppib* has previously been shown to be a suitable housekeeping gene, and similar to recent work from our laboratory (31), in the current study we found that raw CT values were not different between groups. Primer sequences for *Gdf15* were as follows: forward: 5′- GAGCTACGGGGTCGCTTC-3′ and reverse: 5′- GGGACCCCAATCTCACCT-3′. Primer sequences for *Ppib* were as follows: forward: 5′- GGAGATGGCACAGGAGGAA-3′ and reverse: 5′- GCCCGTAGTGCTTCAGCTT-3′.

### Liver and Muscle Glycogen

Glycogen concentrations were measured in the liver and triceps as described recently by Schaubroeck et al. (32). Briefly, tissue was homogenized in 0.5 M NaOH followed by heating at 100°C for 30 min with periodic mixing. Na_2_SO_4_ and ethanol were added to the tissue homogenate, and samples were centrifuged at 2,000 *g* for 10 min to precipitate glycogen and then resuspended in ddH_2_O. Sulphuric acid and phenol were added to 50 μL of sample and the reaction proceeded for 30 min. Samples were then transferred to a 96-well plate, and absorbance was read at 488 nM.

### Statistical Analysis

Statistical tests were completed using GraphPad Prism v.9.0 (GraphPad Software, La Jolla, CA). Differences between groups in the study examining the effects of postexercise feeding were analyzed by one-way ANOVA followed by a Tukey post hoc analysis. If data were not normally distributed, a Kruskal-Wallis test followed by a Dunn’s post hoc was used. For the GDF15^−/−^ experiment, data were analyzed using a group by genotype two-way ANOVA followed by a Tukey post hoc analysis. Pearson (normally distributed) or Spearmen’s rank correlation (not normally distributed) coefficient analyses were used to examine associations between serum GDF15 and circulating metabolites and hormones. Data are presented as means ± SD, and individual data points are shown when possible. A relationship was considered significant when *P* < 0.05.

## RESULTS

### Food Restriction Does Not Impact Exercise-Induced Increases in Serum GDF15 in Male Mice

To determine if postexercise feeding impacts exercise-induced increases in GDF15, mice were provided with chow or had food withheld following exercise. Mice ran for ∼107 ± 19 min (mean ± SD) with ∼61% being able to complete the full 2 h. As expected, liver glycogen levels were ∼80% lower immediately following exercise, compared to sedentary (*P* < 0.0001) and exercise-chow (*P* < 0.0001), and were greater in exercise-chow (*P* = 0.0088) compared to exercise-FW (Table 1). Similar to the liver, glycogen was reduced ∼50% (*P* = 0.0016) in triceps muscle immediately postexercise compared to sedentary mice, whereas exercise-chow had greater glycogen concentrations compared to exercise-0h (*P* < 0.0001) and exercise-FW groups (*P* = 0.0324) (Table 1).

**Table 1.** Postexercise nutrition impacts liver glycogen concentrations in male mice

	Sedentary	EX-0h	EX-Chow	EX-FW
Liver, µg/mg wt/wt	3.03 ± 2.05 (18)	0.51 ± 0.11* (17)	9.22 ± 6.05† (9)	0.68 ± 0.16 (8)
Triceps, µg/mg wt/wt	1.17 ± 0.50 (17)	0.52 ± 0.31* (17)	1.56 ± 0.76† (8)	0.89 ± 0.29 (8)

Data are presented as means ± SD with the numbers in parentheses indicating the number per group. Male C57BL/6J mice ran for ∼2 h or until exhaustion at 70% maximum running speed at the beginning of their dark cycle. Liver and triceps were harvested immediately after exercise (EX-0h) or 3 h after exercise with ad libitum access to chow (EX-Chow) or with food withheld (EX-FW) and analyzed for glycogen concentrations. For liver, data were analyzed using a Kruskal-Wallis test followed by a Dunn’s post hoc. For triceps, a one-way ANOVA was used followed by a Tukey post hoc test. Liver: *significantly different (*P* < 0.0001) than sedentary and exercise-chow; †significantly different (*P* = 0.0088) than EX-FW. Triceps: *significantly different than sedentary (*P* = 0.0016); †significantly different than EX-0h (*P* < 0.0001) and EX-FW (*P* = 0.0324).

**Figure 1.**
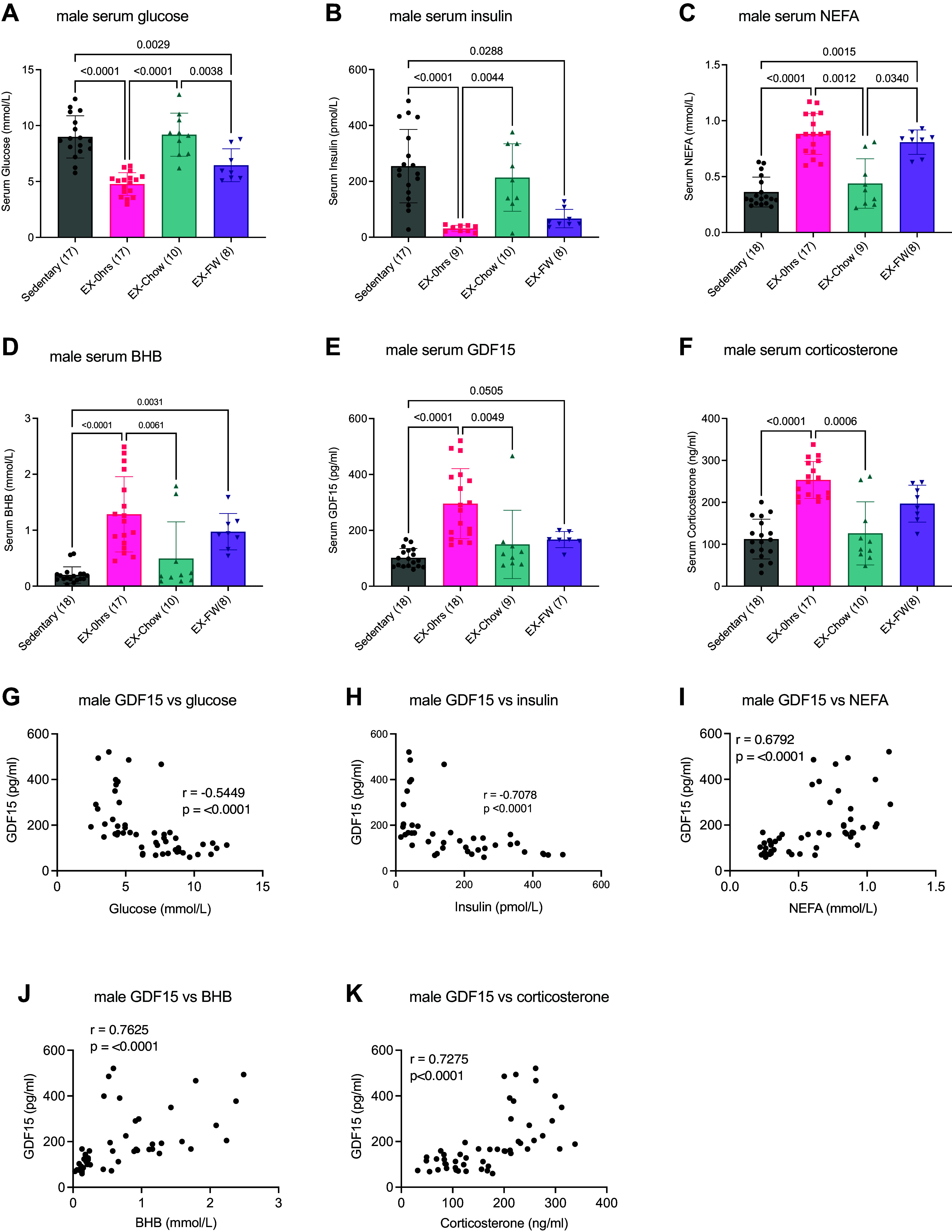
Exercise-induced increases in serum growth differentiation factor 15 (GDF15) are not impacted by postexercise nutrition but are associated with indices of glucose and lipid metabolism in male mice. Male C57BL/6J mice ran for ∼2 h or until exhaustion at 70% maximum running speed at the beginning of their dark cycle. Serum was harvested immediately after exercise (EX-0h) or 3 h after exercise with ad libitum access to chow (EX-Chow) or with food withheld (EX-FW). Serum was analyzed for glucose (*A*), insulin (*B*), nonesterified fatty acids (NEFA; *C*), beta-hydroxybutyrate (BHB; *D*), GDF15 (*E*), and corticosterone (*F*), and associations between GDF15 and glucose (*G*), insulin (*H*), NEFA (*I*), BHB (*J*), and corticosterone (*K*) were determined. Data are presented as means ± SD with individual data points shown. Numbers in parentheses below the *x*-axis represent the number per group in *A*–*F*. Data in *A* were analyzed by one-way ANOVA followed by a Tukey post hoc test when significance was found. Data in *B*–*F* were analyzed by a Kruskal-Wallis test followed by a Dunn’s post hoc analysis. Bars connected by lines are significantly different at the *P* value shown. Associations between GDF15 and glucose (*G*) were analyzed using Pearson correlational analysis, and GDF15 and insulin (*H*), NEFA (*I*), BHB (*J*), and corticosterone (*K*) were analyzed by Spearman correlation. *Insets*: *r* and *P* values for each relationship.

As shown in Fig. 1*A*, serum glucose levels were lower immediately postexercise (*P* < 0.0001) and in exercise-FW (*P* = 0.0029) compared to sedentary mice, while glucose was higher in mice given chow compared to mice immediately after exercise (*P* < 0.0001) or mice who had food withheld (*P* = 0.0038). Serum insulin was lower immediately after exercise, compared to sedentary (*P* < 0.0001) and exercise-chow (*P* = 0.0044), with exercise-FW being lower than sedentary (*P* = 0.0288).

Serum NEFA was higher immediately after exercise (*P* < 0.0001) and in exercise-FW (*P* = 0.0015) compared to sedentary mice, with exercise-chow (*P* = 0.0061) lower than exercise-0h (*P* = 0.0012) and exercise FW (*P* = 0.034). Similar to NEFA, serum BHB was greater immediately after exercise compared to sedentary (*P* < 0.0001) and exercise-chow (*P* = 0.0134), with exercise mice who had food withheld displaying elevations in BHB compared to sedentary (*P* = 0.0031) (Fig. 1*D*).

Serum concentrations of GDF15 were approximately threefold greater in mice immediately postexercise compared to sedentary (*P* < 0.0001) and exercise-chow mice (*P* = 0.0049), and there was a trend (*P* = 0.0505) for serum GDF15 to be elevated in exercise-FW compared to sedentary mice (Fig. 1*E*). Similar to GDF15, serum corticosterone concentrations were higher immediately after exercise compared to sedentary (*P* < 0.0001) and exercise-chow (*P* = 0.0006) (Fig. 1*F*). Collectively, these findings provide evidence that exercise-induced increases in GDF15, unlike markers of carbohydrate and lipid metabolism such as glucose, insulin, NEFA, and BHB, are largely unresponsive to alterations in postexercise nutrient availability in male C57BL/6J mice.

### Changes in Serum GDF15 Are Associated with Indices of Carbohydrate and Lipid Metabolism

Although withholding food following exercise did not prolong increases in GDF15, we sought to explore associations between circulating GDF15 and indices of carbohydrate and lipid metabolism. Using correlational analyses, we found negative associations between GDF15 and glucose (*P* < 0.0001, Fig. 1*G*) and insulin (*P* < 0.0001, Fig. 1*H*)) and positive correlations between GDF15 and NEFA (*P* < 0.0001, Fig. 1*I*), BHB (*P* < 0.0001, Fig. 1*J*), and corticosterone (*P* < 0.0001, Fig. 1*K*) in male mice.

### Food Restriction Prolongs Exercise-Induced Increases in Serum GDF15 in Female Mice

Given the well-characterized sex differences in the acute metabolic response to exercise and exercise recovery (33–36), we repeated the experiments using female C57BL/6J mice. Mice ran for ∼95 ± 31 min (mean ± SD) with 50% of the mice being able to complete the full 2-h exercise bout. Liver glycogen concentrations were approximately five- to sevenfold greater (*P* < 0.0001) in exercise-chow compared to all other groups (Table 2). Glycogen concentrations were elevated by ∼70% in the triceps from mice provided with chow compared to exercise-0h mice (*P* = 0.0353).

**Table 2.** Postexercise nutrition impacts liver glycogen concentrations in female mice

	Sedentary	EX-0h	EX-Chow	EX-FW
Liver, µg/mg wt/wt	1.70 ± 0.57 (8)	0.79 ± 0.17 (8)	7.78 ± 4.07* (8)	1.04 ± 0.39 (8)
Triceps, µg/mg wt/wt	0.60 ± 0.16 (7)	0.39 ± 0.13 (7)	0.65 ± 0.22* (7)	0.49 ± 0.13 (8)

Data are presented as means ± SD with the numbers in parentheses indicating the number per group. Female C57BL/6J mice ran for ∼2 h or until exhaustion at 70% maximum running speed at the beginning of their dark cycle. Liver and triceps were harvested immediately after exercise (EX-0h) or 3 h after exercise with ad libitum access to chow (EX-Chow) or with food withheld (EX-FW) and analyzed for glycogen concentrations. Data were analyzed using a one-way ANOVA followed by a Tukey post hoc test. Liver: *significantly different (*P* < 0.0001) than all other groups. Triceps: *significantly different (*P* = 0.0353) than EX-0h.

**Figure 2.**
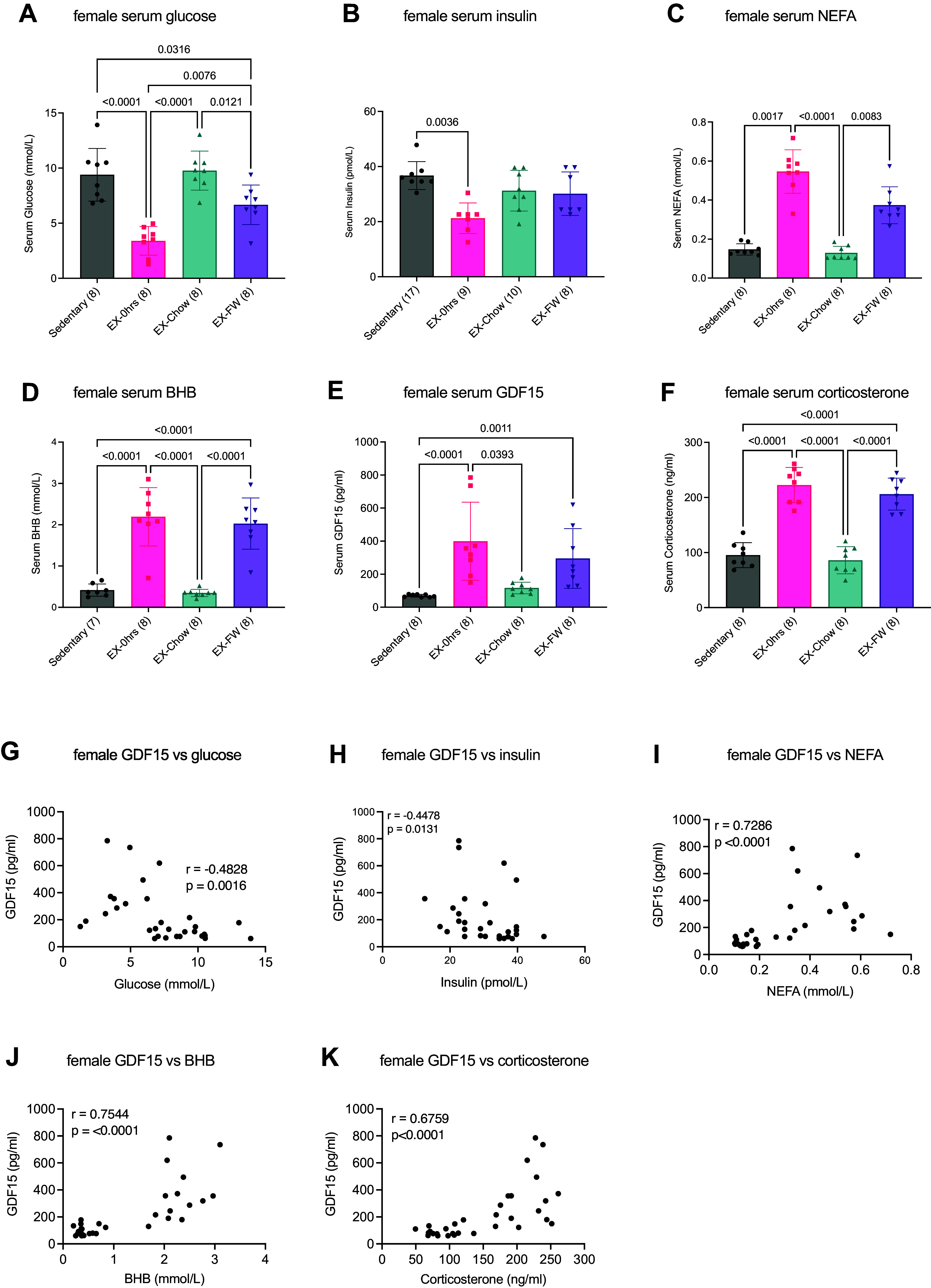
Exercise-induced increases in serum growth differentiation factor 15 (GDF15) are maintained when food is withheld and are associated with indices of glucose and lipid metabolism in female mice. Female C57BL/6J mice ran for ∼2 h or until exhaustion at 70% maximum running speed at the beginning of their dark cycle. Serum was harvested immediately after exercise (EX-0h) or 3 h after exercise with ad libitum access to chow (EX-Chow) or with food withheld (EX-FW). Serum was analyzed for glucose (*A*), insulin (*B*), nonesterified fatty acids (NEFA; *C*), beta-hydroxybutyrate (BHB; *D*), GDF15 (*E*), and corticosterone (*F*), and associations between GDF15 and glucose (*G*), insulin (*H*), NEFA (*I*), BHB (*J*), and corticosterone (*K*) were determined. Data are presented as mean ± SD with individual data points shown. Numbers in parentheses below the *x*-axis represent the number per group in *A*–*F*. Data in *A* and *D*–*F* were analyzed by one-way ANOVA followed by a Tukey post hoc test when significance was found. Data in *B* and *C* were analyzed by a Kruskal-Wallis test followed by a Dunn’s post hoc analysis. Bars connected by lines are significantly different at the *P* value shown. Associations between GDF15 and glucose (*G*), BHB (*J*), and corticosterone (*K*) were analyzed using Pearson correlational analysis, while associations between GDF15 and insulin (*H*) and NEFA (*I*) were analyzed by Spearman correlation. *Insets*: *r* and *P* values for each relationship.

As shown in Fig. 2*A*, serum glucose was lower immediately after exercise compared to sedentary (*P* < 0.0001), exercise-chow (*P* < 0.0001), and exercise-FW (*P* = 0.0076) groups. Serum glucose concentrations were higher in mice provided with chow compared to mice who had food withheld following exercise (*P* = 0.0121). Serum insulin concentrations were lower in mice immediately following exercise compared to sedentary (Fig. 2*B*, *P* = 0.0036).

Circulating NEFA concentrations were elevated immediately postexercise compared to sedentary (*P* < 0.0017) and exercise-chow groups (*P* < 0.0001) with NEFA being greater in mice who had food withheld compared to mice given chow following exercise (*P* = 0.0083) (Fig. 2*C*). Serum BHB was greater (*P* < 0.0001) in mice immediately postexercise and in exercise-FW compared to sedentary and exercise-chow (*P* < 0.0001) groups (Fig. 2*D*).

GDF15 concentrations were approximately fourfold higher immediately postexercise compared to sedentary (*P* < 0.0001) and exercise-chow (*P* = 0.0393) mice, and GDF15 remained greater in mice who had food withheld (*P* = 0.0011) compared to sedentary animals (Fig. 2*E*). Circulating corticosterone concentrations were greater (*P* < 0.0001) immediately following exercise and in exercise-fasted mice compared to the other groups (Fig. 2*F*).

As with male mice, we examined associations between serum GDF15 and circulating markers of glucose and lipid metabolism. We found a negative correlation between GDF15 and glucose (*P* = 0.0016, Fig. 2*G*) and insulin (*P* = 0.0131) and positive correlations between GDF15 and NEFA (*P* = <0.0001, Fig. 2*I*), BHB (*P* < 0.0001, Fig. 2*J*), and corticosterone (*P* < 0.0001, Fig. 2*K*).

### Exercise-Induced Increases in *Gdf15* mRNA Are Largely Not Impacted by Postexercise Nutrition

**Figure 3.**
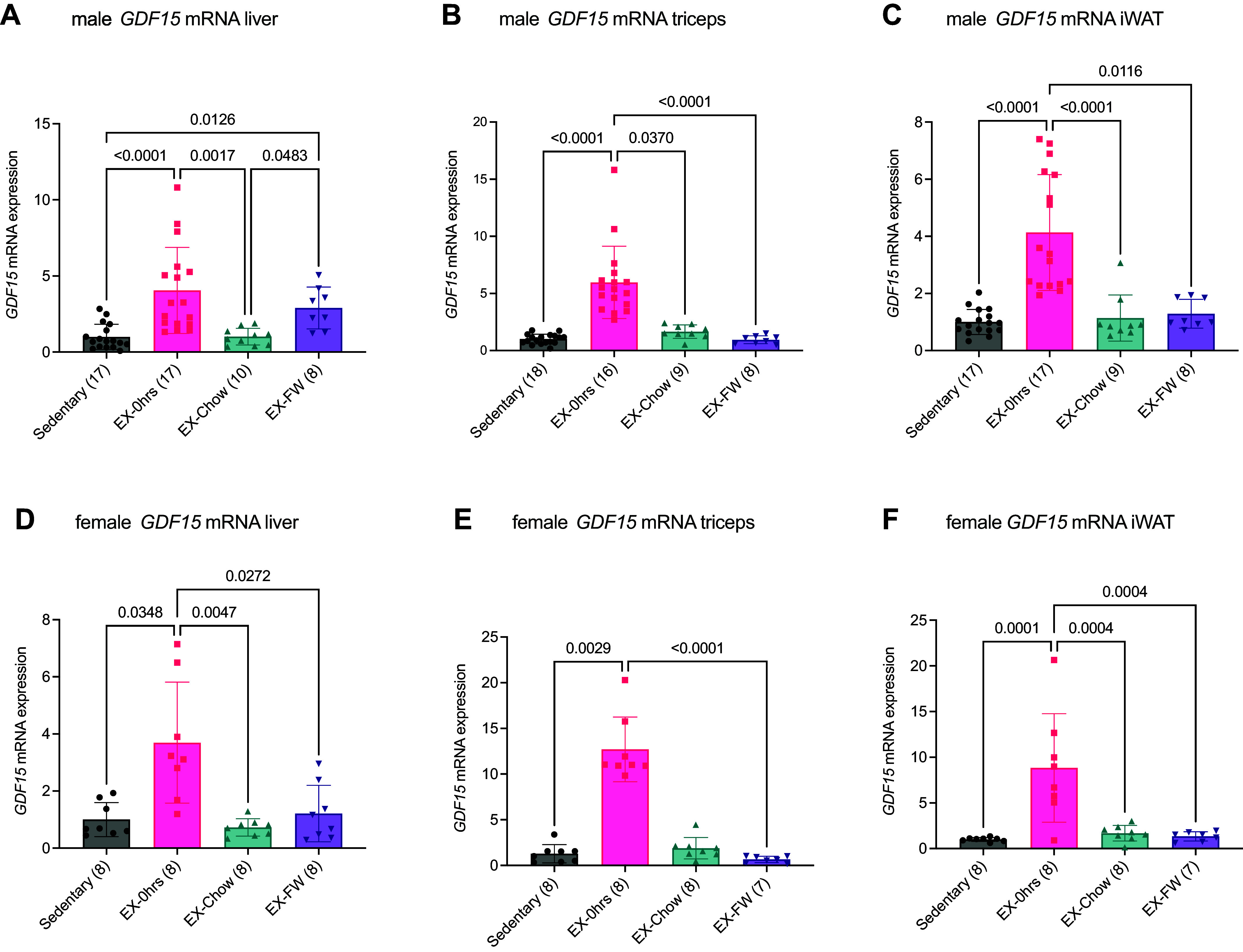
Exercise-induced increases in growth differentiation factor 15 (*GDF15*) mRNA expression are not impacted by postexercise nutrition. Male (*A*–*C*) and female (*D*–*F*) C57BL/6J mice ran for ∼2 h or until exhaustion at 70% maximum running speed at the beginning of their dark cycle. Serum was harvested immediately after exercise (EX-0h) or 3 h after exercise with ad libitum access to a chow diet (EX-Chow) or with food withheld (EX-FW). Liver (*A* and *D*), triceps (*B* and *E*), and inguinal white adipose tissue (iWAT; *C* and *F*) were analyzed for *GDF15* mRNA. Data are presented as means ± SD with individual data points shown. Numbers in parentheses below the *x*-axis represent the number per group. Data were analyzed by a Kruskal-Wallis test followed by a Dunn’s post hoc in *A*–*E* and a one-way ANOVA followed by a Tukey post hoc test in *F*. Bars connected by lines are significantly different at the *P* value shown.

Given the effect of withholding food on serum GDF15, especially in female mice, we next assessed *Gdf15* mRNA expression in tissues that we (13, 16) and others (14, 15) have shown to be responsive to exercise. In liver from male mice, *Gdf15* mRNA expression was approximately fourfold greater immediately postexercise compared to sedentary (*P* < 0.0001) and exercise-chow groups (*P* = 0.0017) and remained higher in exercise-FW compared to sedentary (*P* = 0.0483) (Fig. 3*A*). In triceps muscle from male mice, *GDF15* mRNA expression was approximately fivefold greater immediately following exercise compared to sedentary (*P* < 0.0001), exercise-chow (*P* = 0.0370, and exercise-FW mice (*P* < 0.0001) (Fig. 3*B*). In iWAT from male mice, *GDF15* mRNA was approximately fourfold higher immediately following exercise compared to sedentary (*P* < 0.0001), exercise-chow (*P* < 0.0001), and exercise-FW (*P* = 0.0116) animals (Fig. 3*C*).

Similar patterns of *Gdf15* mRNA expression were seen in tissues from female mice (Figs. 3, *D*–*F*). In liver, *Gdf15* mRNA was elevated approximately fourfold immediately postexercise compared to sedentary (*P* = 0.0348), exercise-chow (*P* = 0.0047), and exercise-FW mice (*P* = 0.0272) (Fig. 3*D*). Likewise in triceps muscle *Gdf15* mRNA expression was elevated ∼12-fold immediately after exercise compared to sedentary (*P* = 0.0029) and exercise-FW mice (*P* < 0.0001) (Fig. 3*E*). *Gdf15* mRNA in iWAT was approximately eightfold greater immediately after exercise versus sedentary (*P* = 0.0001), exercise-chow (*P* = 0.0004), and exercise-FW (*P* = 0.0004) conditions (Fig. 3*F*). Taken together, these findings, at least in female mice, provide evidence that the prolonged elevation in GDF15 following exercise in mice that had food withheld is not paralleled by similar changes in *Gdf15* mRNA expression in multiple exercise responsive tissues.

### Exercise-Induced Changes in Indices of Glucose and Lipid Metabolism Are Intact in GDF15^−/−^ Mice

**Figure 4.**
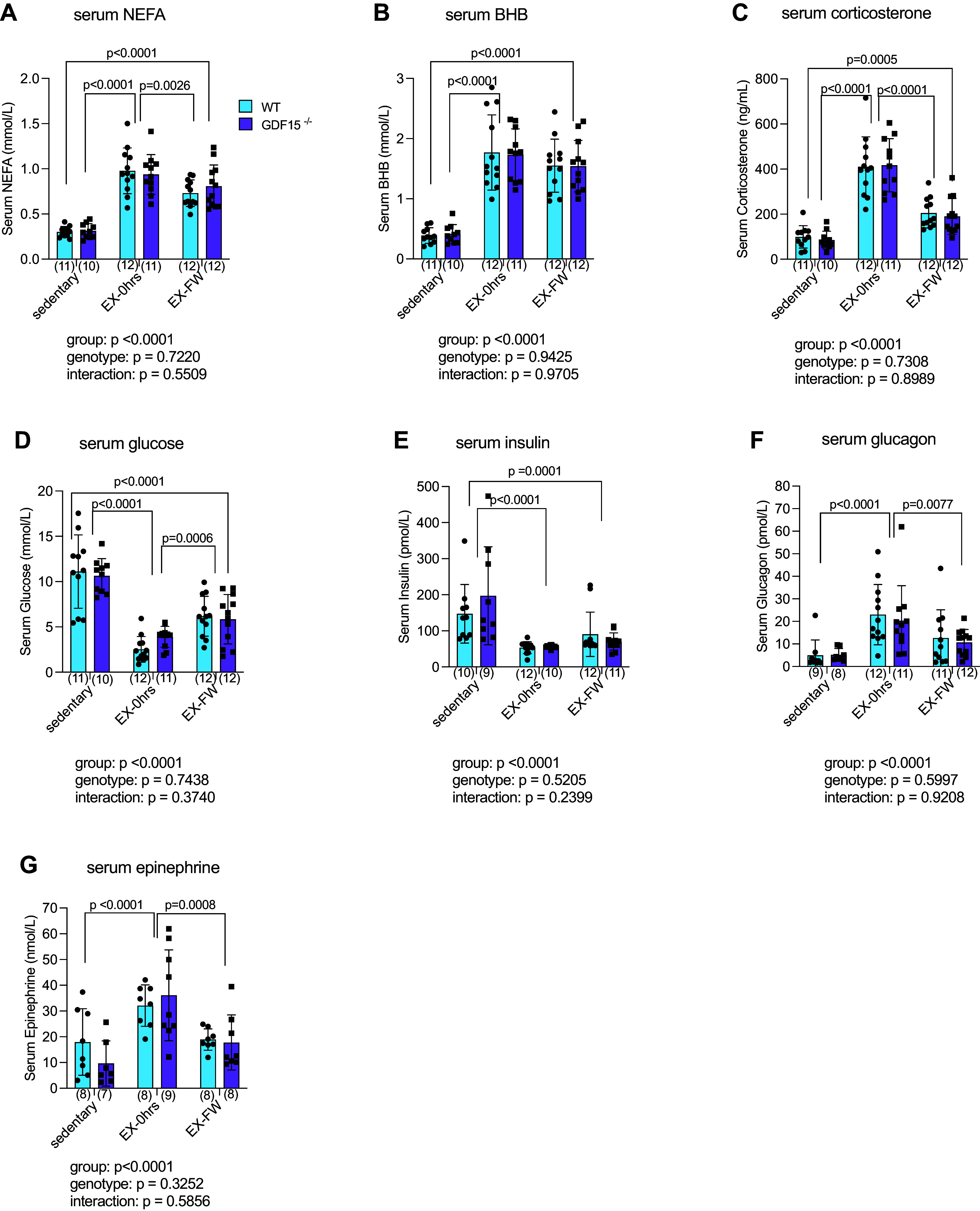
Exercise-induced increases in serum corticosterone (*A*), nonesterified fatty acids (NEFA; *B*), beta-hydroxybutyrate (BHB; *C*), glucose (*D*), insulin (*E*), glucagon (*F*), and epinephrine (*G*) are not different in wild-type (WT) and growth differentiation factor 15-deficient (GDF15^−/−^) mice. Male and female mice ran for 2 h or until exhaustion at 70% of maximum running speed at the beginning of their dark cycle. Serum was harvested immediately (EX-0h) or 3 h after exercise in mice that had food withheld (EX-FW). Data are presented as means ± SD with individual data points shown. Numbers below the *x*-axis in parentheses represent the number per group. Data were analyzed by a two-way (genotype × group) ANOVA followed by a Tukey post hoc analysis. Groups, irrespective of genotype connected by lines are significantly different at the *P* value shown. *Inset*s: *P* values for main (group, genotype) and interaction effects.

Having shown associations between GDF15 and circulating markers of carbohydrate and lipid metabolism, we next wanted to determine the causality of these relationships. Littermate wild-type and GDF15^−/−^ mice (∼10–12 wk of age) ran on a motorized treadmill or remained sedentary, and tissues were harvested either immediately or 3 h following exercise, during which time mice were given access to water but not food. The distance [WT: 2,425 ± 499 m; knockout (KO): 2,416 ± 516; *P* = 0.95] and time (WT: 105 ± 18 min; KO: 112 ± 15; *P* = 0.15) run was not different between genotypes. Given that associations between GDF15 and NEFA/BHB and corticosterone were similar between sexes, both male (M) and female (F) mice were used and data pooled for this experiment (M/F for WT sedentary: 5/6; WT exercise-0h: 6/6; WT exercise-FW: 6/6; GDF15^−/−^ sedentary: 6/4; GDF15^−/−^ exercise-0h: 7/5; and exercise-FW 6/6). As before, an acute bout of exercise elevated serum GDF15 concentrations in WT mice (sedentary: 53 ± 13; immediately postexercise: 122 ± 61 pg/mL; *P* = 0.0014). Confirming the genotype of the mice, serum GDF15 was not detectable in GDF15^−/−^ animals. There was an effect of group (*P* < 0.0001) on fatty acids, with NEFA being greater (*P* < 0.0001) immediately and 3 h following exercise compared to sedentary control. NEFA was also higher (*P* = 0.0026) immediately, compared to 3 h postexercise (Fig. 4*A*). Similar to NEFA, there was a main effect of group (*P* < 0.0001) on serum BHB with concentrations being greater (*P* < 0.0001) in both exercise groups compared to sedentary control (Fig. 4*B*). There was a main effect of group (*P* < 0.0001) on corticosterone with corticosterone immediately postexercise being greater (*P* < 0.0001) than the other two groups (Fig. 4*C*). Moreover, corticosterone was greater (*P* = 0.0005) in exercised mice that had food withheld compared to sedentary animals. Serum glucose concentrations were lower (*P* < 0.0001) in both exercise groups compared to sedentary controls, and glucose was lower immediately following exercise (*P* = 0.0006) compared to mice 3 h following exercise (Fig. 4*D*).

In contrast to our hypothesis, we did not see an effect of genotype on exercise-induced increases in corticosterone, NEFA, or BHB. To determine if this could be due in part to compensatory changes in other mediators of carbohydrate and lipid metabolism, we measured circulating concentrations of insulin (37), glucagon (38), and epinephrine (39), well-established endocrine regulators of fuel metabolism. As shown in Fig. 4*E*, there was a main effect of the grou*p* on serum insulin with both exercise groups being lower (*P* < 0.0001 exercise-0h; *P* = 0.0001 exercise-FW) than sedentary controls. In contrast to insulin, serum glucagon was greater in exercise-0h compared to sedentary (*P* < 0.0001) and exercise-FW (*P* = 0.0077) groups (Fig. 4*F*). Epinephrine was greater immediately postexercise compared to mice in the sedentary (*P* < 0.0001) and exercise-FW (*P* = 0.0008) groups (Fig. 4*G*). In addition to the pooled analysis, we also analyzed the data within each individual sex and found similar effects of group, but not genotype, in male (Supplemental Fig. S1; see https://doi.org/10.6084/m9.figshare.27129186) and female (Supplemental Fig. S2; see https://doi.org/10.6084/m9.figshare.27129216) mice. Taken together, these findings provide evidence that the lack of a genotype effect is not an artifact of compensatory changes in insulin, glucagon, or epinephrine.

## DISCUSSION

GDF15 is increased following acute bouts of exercise, an effect that has been demonstrated in preclinical rodent models (13, 14) and in human participants (14–16). Despite these consistent findings, the physiological role of exercise-induced increases in GDF15 is incompletely defined. In the current investigation, we manipulated postexercise nutrient availability and used this as a model to examine relationships between GDF15 and indices of carbohydrate and lipid utilization. Restricting access to food following exercise led to a prolonged elevation in GDF15 in female but not male C57BL/6J mice with GDF15, being elevated ∼225 pg/mL in females and ∼65 pg/mL in males compared to their respective sedentary controls. The relatively transient increase in GDF15 following acute exercise in mice is in contrast to what has been reported in humans where a single bout of exercise has been shown to result in prolonged increases in serum GDF15 (14, 16). Similarly, exercise training also increases circulating GDF15 in humans (40, 41).

Despite subtle differences in the effects of withholding food on serum GDF15, we saw similar associations between GDF15 and serum corticosterone, NEFA, and BHB in both sexes. Although these associations are supportive of our working hypothesis, causality was not born out by studies using GDF15^−/−^ mice. In these experiments, exercise-induced increases in corticosterone, NEFA, and BHB were not different between wild-type and GDF15^−/−^ mice. Importantly, the lack of a genotype effect did not seem to be explained by compensatory changes in other well-characterized endocrine regulators of glucose and lipid metabolism such as insulin, glucagon, and epinephrine. Similar to our findings exploring the role of GDF15 in mediating the acute effects of exercise on circulating metabolites and hormones, Gil et al. (42) reported that acute exercise-induced increases in the expression of PPARγ coactivator 1-alpha, a master regulator of mitochondrial biogenesis (43), was not different in skeletal muscle from wild-type and GDF15^−/−^ mice. This study is difficult to interpret however as the acute bout of exercise that was employed did not lead to increases in circulating concentrations of GDF15.

In contrast to the present study, the knockdown of GDF15 in the liver attenuated the effects of long-term fasting or a ketogenic diet on increases in serum ketone bodies (25). It could be that GDF15 plays a more important role in the regulation of lipid utilization in conditions of more severe metabolic challenges, where the rise in GDF15 would be expected to be greater and/or for a longer duration. In regards to the former, both long-term fasting, i.e., 24 h (25), and the consumption of a ketogenic diet (44) resulted in similar absolute serum GDF15 concentrations as seen in the current investigation with exercise, ∼200–500 pg/mL. Given this, the duration of the increase in GDF15 may be the more important factor in regard to the role that this hormone plays in lipid utilization. Presumably, both longer term fasting and ketogenic diets lead to a more prolonged increase in GDF15 when compared to the transient increase seen in the current study with exercise.

Recent work has demonstrated that GDF15 is both sufficient to increase corticosterone and necessary for increases in corticosterone induced by infection or chemical toxins in mice (17). Although we demonstrated a clear association between serum GDF15 and corticosterone, there were no genotype differences in the response to exercise. This is somewhat consistent with recent work demonstrating that the peak in serum cortisol with exercise preceded that of GDF15 in human participants (16). It could be that circulating GDF15 concentrations do not reach high enough levels during exercise to trigger increases in corticosterone. In this regard infectious and toxic agents increase GDF15 to several nanograms per milliliter (17), whereas levels reached following exercise are ∼5–10 times less. Similarly, the chemogenetic activation of GFRAL^+^ neurons also leads to robust increases in serum corticosterone in mice (45).

In addition to the findings of the current study examining hormonal and metabolic endpoints, it would also appear that the absence of GDF15 signaling does not impact behavioral readouts following exercise. For example, neither food intake nor subsequent voluntary wheel running behavior was different in wild-type and GFRAL-deficient mice following an acute bout of treadmill running (14). In contrast, endurance exercise performance is enhanced in tumor-bearing mice treated with GDF15 neutralizing antibodies (46). These findings, in combination with the current results, highlight the elusive nature of GDF15 in the context of exercise, especially in otherwise healthy animals.

While speculative at this juncture, it has been proposed that exercise-induced increases in GDF15 could play a role in the anti-inflammatory effects of exercise (47). Prior work has previously shown that an acute bout of intensive treadmill exercise causes a shift in the polarization of adipose tissue macrophages from an M1- to more of an M2-like phenotype (48). If GDF15 were to be mediating this effect it would be independent of corticosterone and/or epinephrine as exercise-induced increases in these hormones, which have noted anti-inflammatory effects (49, 50), were found to be similar between WT and GDF15^−/−^ mice following exercise. Interestingly, some have suggested a direct effect of GDF15 on promoting macrophage polarization to an M2 phenotype (51, 52). These findings, while intriguing, are difficult to reconcile with the tissue expression profile of GFRAL, which is widely believed to be expressed exclusively in the central nervous system (2–5), although more recent findings have suggested a more widespread pattern of expression (53). As suggested by others (41), GDF15 might also be signaling through a GFRAL-independent mechanism.

A limitation of the present study is that animals were housed at room temperature and not thermal neutrality. This was necessary as a dedicated experimental room maintained at ∼28–30°C was not available for the treadmill exercise experiments. Given the impact of cold on corticosterone (54), and the emerging bidirectional relationship between insulin and GDF15 (16, 41), repeating the experiments described in the current investigation at thermal neutrality would increase the generalizability of our findings.

In summary, we have, for the first time, examined the role of GDF15 in mediating exercise-induced increases in corticosterone and indices of lipid utilization in mice of both sexes. While we found associations between GDF15 and the aforementioned endpoints, these associations were not causal and might perhaps suggest that exercise-induced increases in GDF15 might be part of a generalized stress response instead of a bonafide mediator of the acute effects of exercise on fuel metabolism. The findings of the current investigation highlight the elusive role that GDF15 might play in the physiological response to exercise.

## DATA AVAILABILITY

Data will be made available upon reasonable request.

## SUPPLEMENTAL MATERIAL

10.6084/m9.figshare.27129186 Supplemental Fig. S1: https://doi.org/10.6084/m9.figshare.27129186.

10.6084/m9.figshare.27129216 Supplemental Fig. S2: https://doi.org/10.6084/m9.figshare.27129216.

## GRANTS

This work was supported by a project grant from the Canadian Institutes of Health Research (PJT No. 178032 to D.C.W).

## DISCLOSURES

No conflicts of interest, financial or otherwise, are declared by the authors.

## AUTHOR CONTRIBUTIONS

M.A., B.J.B., and D.C.W. conceived and designed research; M.A., B.J.B., S.J., A.B., M.A., S.T., K.E., and K.D.M. performed experiments; M.A., B.J.B., S.J., and A.B. analyzed data; M.A., B.J.B., and D.C.W. interpreted results of experiments; M.A., B.J.B., and D.C.W. prepared figures; M.A., B.J.B., and D.C.W. drafted manuscript; M.A., B.J.B., S.J., A.B., M.A., S.T., K.E., K.D.M., and D.C.W. edited and revised manuscript; M.A., B.J.B., S.J., A.B., M.A., S.T., K.E., and K.D.M. approved final version of manuscript.
